# Mr. Hill, on the Diseases of the Bones

**Published:** 1802-05

**Authors:** George Nesse Hill

**Affiliations:** Chester


### To Dr. BRADLEY.

Sir,

If the following Obſervations be worth inſertion in your Journal, be pleaſed, at your convenience, to give them a place therein, and you will additionally oblige,

Sir, your obliged friend,

*Cheſter, Nov.* 30, 1801. GEORGE NESSE HILL.

---

Notwithstanding the very improved ſtate of Oſteological knowledge, ſince the great advancement made towards aſcertaining the laws which govern the abſorbent ſyſtem, yet much undoubtedly remains to be done on this highly important inexhauſtible ſubject. The diſeaſes of the bones are, perhaps, moſt of them well underſtood, and commonly treated with ſucceſs, yet there is one occurring ſometimes which does not ſeem to have fully ſhared in this ſucceſs; to this I wiſh for a few moments to ſolicit attention, with the hope that what may now be advanced will at leaſt tend to bring forward uſeful obſervations from more experienced practitioners. There can ſcarce be a Surgeon whoſe practice has not afforded him inſtances of thickening or enlargement of the bones, attended with more or leſs of pain, impediment of motion, deformity, neighbouring diſeaſe, general diſeaſe, and ſometimes ultimately death. The univerſal influence and wonderful powers of the abſorbent ſyſtem in all the functions of the animal œconomy are in our day ſo well known and ſo frequently illuſtrated, that it may ſafely be ſaid the great improvements our Art has acquired in modern times, owe their riſe in a conſiderable degree to this ſource; it may alſo, without heſitation be averred, that he is the moſt ſucceſsful practitioner in medicine, who moſt clearly comprehends their laws, offices, and extenſive powers, and who has cultivated this branch of knowledge with the moſt aſſiduity; ſtill it cannot be denied that much remains to be done in this extenſive vineyard, and that there is every inviting proſpect of ſucceſs to all who ſhall attempt farther elucidation in ſo uſeful a department of medical ſcience. Many excellent cellent Treatifes have been fent into the world profeffedly on the fubject of ulcers of the legs; and the more voluminous works of authors in general, on Surgery, have always treated this fubject with peculiar attention: Nor without juft reafon has fo much time and pains occupied their abilities, this fountain of human calamity affording fuch numerous ftreams as to conftitute a very large proportion of the objects of chirurgical regard. In what little I have to advance on a matter of fuch confequence and magnitude, I mean to confine myfelf to that fpecies of difeafed bone arifing from redundant depofition of boney matter proceeding from a variety of caufes, as blows, bruifes, obftruction, ftimuli circulating in the fluids, as the venereal virus, caries, and the confequences (fometimes) of amputation. The thickening and confequent disfigurement of bones accompanying ulceration, does not always originate in the bone, but fometimes in its compages; but, I believe, it will be found that the bone is always more or lefs materially concerned whenever its peculiar invefting membrane, the periofteum, is difeafed. The bones have been fo accurately defcribed, as to their general fabric, by our able phyfiologifts, that it would be fuperfluous to fay more on this head, than barely to recall to the minds of thofe who fhall honor thefe remarks with a perufal, that they are compofed of blood veffels, nerves, membranes, fat, lymphatic veffels, and cellular fubftance, all more or lefs firmly united with an hard, earthy, infenfible matter; fo that allowing for this circumftance, and peculiar fituation, thefe blood veffels, nerves, &c. are as capable of being acted upon by common caufes as the fame parts are in any other fituation; hence they may be deranged in all the ordinary modes, and alike influenced by common remedies, allowing for difference of fituation. Offific inflammation muft be treated as mufcular inflammation, although accompanied and followed by peculiar phænomena, the hard refifting earthy matter not fuffering the vafcular parts to diftend as in a mufcle; hence, without the interpofition of fuitable means, deftruction or caries muft follow. But very extenfive and alarming difeafe will take place in bones without caries, every practitioner being daily fenfible that the terms, carious bone and difeafed bone mean two very different things; the former implying the mortification or death of the part, the latter is expreffive of any affection the refult of inflammation, defective abforption, redundant fecretion, periofteal thickening, and a great variety of caufes. It is of difeafed bone, and of that fpecies arifing from redundant fecretion of boney matter, I am now to treat: This affection may be either fimple or compound, as it is, or is not, connected with caries of the bone or ulceration of the contiguous foft parts; a blow a blow on the tibia, or a kick on the ſhin, as it is commonly called, often gives us the firſt kind; whilſt fractures, wounds, ancient ulcers, and the conſequences of amputation, afford the laſt; in both, the effects produced by the ſtate of the ſyſtem are ſimilar: a ſimple blow on a bone ſhall ſometimes be the exciting cauſe of extenſive miſchief; Nature, as it were, takes the alarm, and immediately commences a proceſs exactly ſimilar to that which follows fracture, viz. Firſt, ſecretion of coagulable lymph as a matrix; Secondly, depoſition of boney matter therein, and conſequent tumor, which has ſometimes unhappily been miſtaken for a ſyphilitic affection. A brick-layer's labourer, ſome time ſince, fell from the ſecond ſtory of a new building; he received conſiderable hurt of his left thigh bone, in ſuch a manner as to bruiſe but not to break it; the ſhock and injury were great, but unaccompanied with even abraſion of the ſkin, nor did extenſive inflammation follow; but the man endured unintermitting pain with rapid increaſe of the whole femur from above downwards to the ſpot hurt, and at length total inability to uſe it; he died quite exhauſted. Permiſſion being given to examine it, boney matter was found to have been depoſited in ſuch quantity from the hip joint to the bruiſed part (within a few inches of the condyles) as to have quite ſunk the ſufferer under the inordinate, uſeleſs exertion. Perhaps no hurt, however ſmall, is inflicted on a bone without a greater or leſs degree of this proceſs taking place; exuberant ſecretion is manifeſted in moſt fractures, and after amputation, as already obſerved, it ſometimes cauſes extenſive miſchief and death; this is almoſt to a certainty the caſe where the end of the bone dying is to be thrown off by the efforts of Nature. Abraſion, or ſimple wounds of bones, or of the periosteum, are ſometimes ſucceeded by conſiderable inflammation, the firſt ſtep in the proceſs; the ſecond is depoſition, and this may follow with or without previous loſs of ſubſtance. This redundance muſt be diſtinguiſhed from tumefaction, the effects of defective abſorption occurring in ſcrophula; alſo from obſtruction, the effects of ſyphilitic ſtimulus circulating in the fluids. No ſuperior diſcrimination is neceſſary for this purpoſe, common attention alone being a ſure guide; thus the rule of diagnoſis may be drawn from the abſence of general ſcrophulous or ſyphilitic ſymptoms, from the age, general health of the patient, from a conſideration of the exciting cauſes, as blows, bruiſes, &c. Ruptured lymphatics will ſometimes be the conſequence of great exertions, as in difficult parturition, and ultimately produce incredibly large tumours, *rudis indigestaque moles*; but here the exciting cauſe commonly points out at once the diſtinction where early attention has been paid to the ſymptoms and

 progreſs progrefs of difeafe. Continual depofition and abforption are unceafing proceffes in the animal œconomy; health, therefore, muft of confequence greatly depend upon the due equilibrium being preferved between thefe two grand and conftant operations. That fecretion is conftant and abfolutely indifpenfible is univerfally allowed, how it fhould happen that proportionate abforption fhould have been overlooked on fome occafions is matter of wonder; on this certain fact, notwithftanding, the whole phænomena of fcrophula depends. But to return more directly to the fubject of this paper, we deduce from this obfervation, confirmation of our leading affertion, viz. that redundant fecretion is the caufe of all the evil in contemplation; abforption goes on as ufual, but its agents, the lymphatics, remove only the thinner parts or coagulable lymph, as in forming new bone for the cure, fracture leaving the hard earthy particles to accumulate; hence, in a longer or fhorter period, proportionate pain, fwelling, derangement of periofteum, cellular fubftance, blood veffels and nerves; in fhort, of all the contiguous parts to the integuments: Thefe are the confequences which may be termed local; the general fymptoms are pyrexia, debility, emaciation, the languor of exhauftion, hectic fever, and death, from the emphatical caufe a patient once alleged to me, that all the food and medicines he took went but to nourifh the fwelling. But exclufive of thefe fatal cafes we have the difeafe in a lefs terrific form almoft daily occuring with and without ulceration, efpecially in the leg; the firft and moft fimple form is that arifing from a blow inflicted without the flighteft apparent cuticular injury, the fucceeding tumour affuming the appearance of exoftofis; the fecond is a more diffufed or unequal enlargement accompained with ulceration, in one or more frequently various fpots of the limb; a third may be denominated, that where a blow or bruife has fo fhaken the bone as to caufe the death of a part; here we fhall have a thickening to an amazing extent, proceeding from the exertions of Nature to free the healthy from the deftroyed parts; this fpecies often fucceeds compound fracture, is always accompanied with ulceration, and comprehends thofe which occur fubfequent to amputation, when a portion of the whole divided bone muft be thrown off; thefe three fpecies embrace the whole feries of tumours from redundant fecretion. The primary curative indication is analagous to that indicated in fcrophula, with the difference or diftinction before mentioned, viz. the excitement of the abforbent veffels to a greater force or degree of action; but the ftriking diftinction confifts in the one requiring diminution of the fecreting procefs and the other not. In fcrophula there does not appear to exift inordinate fecretion, tion, but fimply inert or defective abforption; hence the redundance arifes from oppofite caufes, and is attended with a ftriking difference as to general health and appearance; in the inftances which have fallen under my notice, the fufferers have uniformly been of that habit of body which is indicative of an oppofite ftate to the fcrophulous diathefis. Defective fecretion of boney matter we fee illuftrated in rachitis, it alfo fometimes occurs in fractures; I recollect a cafe of fractured humerus in the London Hofpital fome years ago, which from the time of the accident to the end of the period commonly taking place for the reunion of this bone, never manifefted other figns of folidity than the firft ftep, namely, the fecretion of coagulable lymph. Under the idea that the man might be fuffering from a venereal taint, mercury was adminiftered fo as gently to affect the mouth; ftill nothing was gained, Nature being as reluctant in completing the work as before. As a powerful ftimulus to produce complete reftoration, an incifion was made through the foft parts, fufficiently large to expofe the fractured ends of the bone; they were found barely glued together by the coagulable lymph; this was removed, and alfo a very fmall portion of bone, with the faw; the whole afterwards replaced in a proper pofition, an effort appeared foon to be made towards healthy completion of the bufinefs, for at the end of a month re-union more folid than before had taken place, but ftill far from what was abfolutely neceffary to complete cure. Tonics, cold bathing, and every thing in fhort that great ability could fuggeft, were tried to roufe the dormant action of the fecreting veffels, but the neceffary depofition feemed as far off as ever. In this ftate of matters I left the houfe, and did not (through accident) learn what became of the poor unfortunate fufferer. At the very fame period a ftrong lufty man was admitted into the fame Hofpital, with an enlarged thigh from the hip to a little lower than the middle, painful but not inflamed; after increafing to an enormous fize, producing great weaknefs, hectic fever, &c. he died. Examination being permitted, a fmall portion of the bone at the inferior extremity of the tumor was found carious; to throw off which, the whole femur to the carious fpot was covered with boney matter to an amazing thicknefs; the very membranes and mufcles were loaded with fimilar fubftances. Thefe two cafes form a ftriking contraft illuftrating the effects of defective and inordinate fecretion, at the fame time pointing out the line of conduct to be purfued by the Surgeon, never lofing fight of the juft diftinction to be made between redundant fecretion and defective abforption, error in this refpect being the moft dangerous rock on which inadvertence can be driven. In both cafes the ftriking local fymptoms are tume-

 faction, faction, pain, immobility, and earlier or later inflammation with general affection; but in the latter case the symptoms of general affection are strongly indicative of scrophulus habit, whilst in the former they are wholly absent. A just diagnosis being formed, the mode of treatment will be suggested from the history, symptoms, exact nature and situation of the species of disease, viz. to produce such an increased action in the absorbents as shall effectually counteract the inordinate action of the secretory vessels of the part diseased. If the original cause be bruised or hurt bone, without wound or ulceration, and the sufferer be young, that is, under middle age, and possessing a constitution that will admit of bleeding, it may be the first step, local bleeding by leeches, or cupping, will advantageously succeed; frictions and compression of the whole limb alternately will be found powerfully promotive of absorption; internally, gentle emetics, cathartics, and at intervals small doses of mercury, the tinct. digitalis, nitre when it acts by the kidneys, tend to the desired effect: These means, with an abstemious diet, especially with regard to liquids, will commonly succeed; but in every description of case I have seen, a liberal use of the nitric acid has been most eminently succesful, so much so, as to give rise to these desultory remarks. Whatever may be the antivenereal properties of this valuable medicine, it is not for me to attempt to decide; but of its powers to excite extraordinary action of the absorbent system, I can speak with satisfactory confidence; its exhibition in all affections of the bones of the nature here spoken of, has always afforded me pleasure; its power over this highly important part of the system seems second to nothing but mercury. Cutaneous inflammation, excited by rubefacients, as often as the skin will bear it, is a powful auxiliary; and when recourse cannot be had to it exactly over the part affected, it may be used as near thereto as possible, and that with an effect not to be despised, when it is cooly considered that it is from the aggregate effect of a number of remedies (perhaps individually applied but of small importance) that is to give a beneficial result in these cases. Hence the means employed can scarcely be too numerous, provided they all tend to this common centre; nor can they be too perseveringly employed, if complete success is to be the reward: in short, *hic labor hoc opus est*. Compression has evinced its power in promoting absorption in the cases of conical stump, formerly so common; and that pressure increases the action of the lymphatics, is manifest to the most incurious observer. The compress, whatever be its description, should be applied over the whole diseased limb, be the disease situated where it may. For the leg, the laced stocking, when nicely adapted and and made of firm materials, has the advantage over the roller, efpecially to labouring men, who can afford but little time for reft or recumbent pofture. Thefe means, judicioufly applied, will commonly anfwer the defired end; where this is not the cafe, and Nature does not feem to take the hint given her with adequate alacrity, a fontanelle may be advantageoufly added to the curative means. It fhould be placed as near the enlargement as poffible, and be made of a fize adapted to the ftrength of the patient and magnitude of the evil; thus the early and moft fimple form of the difeafe will be fuccefsfully combated. When undue depofition of boney matter and ulceration are combined, the cafe becomes more complex; ftill no caries may exift, difeafed bone, as before remarked, not being always carious bone; thickened periofteum will fometimes occafion much apparent deformity of a limb, and yet the bone be but little or not at all affected, unlefs by neglect or mifmanagement; thefe are the fituations of moft frequent occurrence in practice, and often leave very deformed limbs after the ulceration be healed; fuppofing this part of the bufinefs then to have been completed, fuitable friction, compreffion, and the nitric acid will finifh the cure. When ulceration has been extenfive, with great difcharge and confiderable debility, the acid at the fame time as it excites greater action of the abforbent veffels, gives increafed and healthy tone to the ftomach and whole fyftem, its good effects are powerfully feconded by the occafional exhibition of the oxygenated muriate of potafh. On the fubject of exercife and reft in thickened legs with ulceration, I have found that where the pain was not great, the inflammation not inordinate, nor the fores very extenfive, moderate exercife facilitated reftoration, efpecially where the fubject was accuftomed to hard labor or much motion when in health, a circumftance of habit never to be overlooked. Too great anxiety to keep under or fubdue inflammation will be mifchievous, becaufe without this falutary procefs, in a certain degree, the great bufinefs of abforption would fall fhort of the defired end. But laftly, confiderable difeafe fometimes occurs from the efforts of Nature, to remove even a fmall bit of bone, by no means bearing a proportion to the extent and fituation of the offending body to be removed: Could we but afcertain exactly how much requires removal, and by any means extricate it, the work of the fyftem, as to the getting rid of what is become extraneous, would be thus at once completed; in the efforts to effect this it is that new mifchief is added to the old, the former becoming too often the moft formidable, there being often an amazing rufh of effort (if I may fo term it) to all the neighbouring parts; where it can be done, mechanical chanical aſſiſtance may ſave ſuch a limb from amputation or life from deſtruction; I mean, where the ſituation of the growing miſchief will admit of timely removal of the diſeaſed part, for the exertions of ſeparation are ever accompanied with the ſecretion and depoſition of new boney matter; this deranges as it proceeds, creates new ſymptoms of miſchief extending farther daily, till at length the limb is condemned to removal, or, if that be impracticable or rejected, death, from exhauſtion, ends all.—That theſe are the inevitable conſequences of delay will be readily granted, when it is conſidered that if a portion from the ſurface or middle of a bone is, owing to ſome cauſe or other, to be thrown off, there muſt be a chaſm formed between it and the ſound parts by the neceſſary preliminary action of the abſorbents, in order to detach the now ſuperfluous body; but that this ſalutary proceſs failing to take place, new boney matter is prematurely and redundantly ſecreted, covering the place day after day, all the conſequences already enumerated therefore muſt inevitably follow. Where ſituation of parts and anatomical knowledge will juſtify the operation, the redundant offenſive parts muſt be removed as ſpeedily as poſſible, or Nature will be deprived of that aſſiſtance which Art certainly has it in her power to beſtow; when this is impracticable, all the means before mentioned as promotive of abſorption, during the exfoliating proceſs, muſt be our dependence for expediting the grand buſineſs in view. Should this happily be effected, due ſecretion of healthy particles will ſoon follow, and of conſequence reſtoration, which, with a ſound limb, although not a handſome one, is incomparably preferable to no limb at all. By ſtrict attention to theſe circumſtances as they ſeverally ariſe, Nature will receive that aſſiſtance which it is the proper buſineſs of Surgery to give, and her ill directed unavailing efforts to remove diſeaſe be conducted to an healthy inſtead of a fatal termination. The nitric acid will be found to ſtand in the foremoſt rank of the ſorbentia; ſometimes it diſagrees a little with the bowels, but this circumſtance will be more frequently found to ariſe from irregularities in food than from the medicine; occaſional uſe of columbo root obviates the inconvenience when the acid alone is to blame; ſometimes it paſſes off too freely by urine, therefore ſhould it be determined principally to the bladder, although diuretics powerfully promote abſorption, it may be deſirable to check its effects this way occaſionally, and that is readily done by exhibiting a few doſes of the oxygenated muriate of pot aſh immediately before each doſe of the acid.

